# Industrial-Scale Injection Moulding Validation of Recycled Fiber-Reinforced Polypropylene: Processability and Manufacturing Feasibility

**DOI:** 10.3390/ma19112314

**Published:** 2026-05-30

**Authors:** Elena Picazo Camilo, Raúl Carrillo Beltrán, Griselda Elisabeth Perea Toledo, Francisco Antonio Corpas Iglesias, Vesna Žepič Bogataj, Simon Kotnik, Francisco Javier Iglesias Godino

**Affiliations:** 1 EcoCastulum, Avenida de la Universidad 1, 23700 Linares, Spain; cro@ecocastulum.com (R.C.B.); gept@ecocastulum.com (G.E.P.T.); cto@ecocastulum.com (F.A.C.I.); 2 Department of Chemical, Environmental and Materials Engineering, Higher Polytechnic School of Linares, University of Jaén, Campus Científico-Tecnológico de Linares, 23700 Linares, Spainfigodino@ujaen.es (F.J.I.G.); 3 TECOS, Slovenian Tool and Die Development Centre, Kidričeva Cesta 25, SI-3000 Celje, Slovenia; vesna.zepic.bogataj@tecos.si; 4 Gorenje Gospodinjski Aparati, Partizanska Cesta 12, SI-3320 Velenje, Slovenia; simon.kotnik@gorenje.com

**Keywords:** recycled polypropylene, recycled composites, post-consumer fiber, fiber-reinforced composites, compounding, waste, circular economy

## Abstract

**Highlights:**

- Industrial validation of recycled PP reinforced with natural fibers.
- Robust injection moulding confirmed across multiple industrial sites.
- No equipment modifications required under standard production conditions.
- Industrial-scale processability was successfully demonstrated under real manufacturing conditions.
- Suitable for implementation in real industrial manufacturing environments.

**Abstract:**

This study evaluates the industrial-scale feasibility of injection moulding of a recycled polypropylene composite reinforced with recycled fibers derived from an industrial waste stream. Although previous laboratory-scale research has demonstrated the potential of natural fiber-reinforced thermoplastics, their large-scale industrial implementation remains limited due to uncertainties related to processability, reproducibility, and manufacturing robustness. In this work, the composite material is validated through injection moulding trials carried out in four independent industrial companies located in Andalusia (Spain) and three industrial case studies across different industrial sectors in Slovenia, operating under real production conditions. The extrusion process was characterized in terms of process stability, confirming continuous operation with automated dosing and stable material flow without interruptions under industrial conditions. Injection processing parameters, cycle stability, part quality, and defect formations are also considered important when assessing the manufacturing feasibility. The multi-site validation approach enables the evaluation of reproducibility across different injection moulding systems and mould geometries, providing critical insights into the scalability and technological readiness level of recycled natural fiber-reinforced polypropylene composites. Although direct energy consumption measurements were not systematically recorded, the observed processing stability and cycle repeatability indicate a consistent and energy-efficient operation under industrial processing conditions. The results contribute to bridging the gap between laboratory-scale material development and real industrial implementation.

## 1. Introduction

The transition towards more sustainable materials has accelerated the development of recycled polymer composites in recent years [1,2]. Polypropylene (PP) is one of the most widely produced and used thermoplastic polymers worldwide due to its versatility, low cost, and favorable mechanical and chemical properties [3]. Certain industrial waste streams represent a valuable opportunity for PP recovery and its reintegration into new production cycles. Its presence is particularly significant in high-volume industrial sectors such as household appliances [4], where manufacturing processes generate considerable amounts of PP waste in the form of purges, scraps, and material accumulations. In the case of GORENJE, a leading Slovenian manufacturer of household appliances, these post-industrial residues constitute a potential source of secondary raw materials. Within the framework of the INCIRCULAR project (ID 101114988), these internal material streams have been recovered and reprocessed to obtain recycled polypropylene (rPP), intended for evaluation in new composite material formulations. This industrial-driven valorization strategy represents a key step towards closing the loop in high-volume manufacturing environments, where internal waste streams are often underutilized despite their high potential for material circularity.

In recent years, numerous studies have focused on the development of natural fiber-reinforced polymers [5,6], which have attracted increasing attention due to their lower environmental impact, low density, cost-effectiveness, and acceptable mechanical performance for non-structural applications. In particular, natural fiber-reinforced PP represents a promising alternative to conventional materials for applications in the automotive, construction, and consumer goods sectors, especially when combined with recycled polymer matrices derived from industrial waste streams [7]. Other research efforts have addressed fiber treatment, fiber–matrix compatibility, mechanical performance optimization, and microstructural characterization [8,9,10]. However, most of these studies remain primarily material-centered, focusing on laboratory optimization rather than full process-chain validation under industrial manufacturing conditions [11,12].

Laboratory-scale studies on these composite systems have demonstrated improvements in stiffness and specific strength, as well as the possibility of tailoring material properties through variations in fiber content and processing conditions. However, most of these investigations are conducted under controlled laboratory conditions using virgin PP, typically employing compression moulding or small-scale injection series, where process variability is limited, and parameters are tightly controlled [13,14]. Consequently, the transferability of these results to industrial-scale injection moulding environments remains uncertain, particularly when recycled matrices and secondary fibers are used simultaneously [15].

Despite these advances, the large-scale industrial implementation of recycled PP composites reinforced with recycled natural fibers remains limited. One of the main challenges lies in the gap between laboratory characterization and industrial processability. Mechanical performance measured under controlled conditions does not necessarily ensure stable behavior during industrial injection moulding. Factors such as the thermal resistance of natural fibers, fiber breakage during plastification, rheological variability of recycled matrices, moisture sensitivity, and machine-to-machine differences may significantly affect process stability and final part quality [16,17]. In addition, industrial production environments introduce further complexities, including cycle time constraints, mould design variability, and machine-specific processing windows.

Another critical aspect that remains insufficiently addressed in the literature is reproducibility across different manufacturing environments. Demonstrating that a recycled fiber-reinforced material can be successfully processed in a single laboratory setup does not guarantee its robustness across different industrial facilities operating with diverse injection moulding machines, mould geometries, and processing strategies. From a technology transfer perspective, multi-site validation is essential to increase industrial confidence and raise the technology readiness level (TRL) of emerging sustainable materials. This multi-site industrial validation approach is still rarely reported in the field of recycled polymer composites, despite being essential for real deployment and scale-up.

In this context, the validation of recycled PP reinforced with natural fibers under real industrial injection moulding conditions becomes a crucial step toward market adoption. It is necessary to assess process stability, defect formation during processing, and the retention of mechanical properties in the final injected parts.

The objective of this work is to evaluate the industrial-scale injection moulding feasibility of a recycled polypropylene composite reinforced with natural fibers. To this end, a recycled PP matrix reinforced with post-consumer cellulose fibers was selected in order to assess the potential of integrating secondary polymer resources into high-value composite materials for industrial applications. The material, previously characterized at laboratory scale [4], is validated through injection trials conducted in seven independent industrial companies located in Southern Spain and Slovenia, both considered less-developed regions. Processing parameters, manufacturing stability, part quality, and post-injection material properties were analyzed to evaluate reproducibility and robustness across different production environments. The novelty of this work lies in the combination of (i) a fully recycled polymer–fiber system, (ii) industrial waste valorization within a real manufacturing context, and (iii) a multi-site industrial validation strategy, which together enable a comprehensive assessment of technology readiness beyond laboratory-scale studies. By bridging the gap between laboratory-scale development and real manufacturing conditions, this study aims to provide an assessment of the industrial feasibility of this type of material.

## 2. Materials and Methods

### 2.1. Matrix and Fibers

#### 2.1.1. Recycled Polypropylene (rPP)

The composite material used in this study consisted of recycled polypropylene (rPP) matrix filled with 30 wt.% of talc and CaCO_3_ mineral mix, obtained in milled form from post-industrial waste generated during the manufacturing of household appliances at GORENJE factory (Velenje, Slovenia). Due to the extensive use of PP in various components of these products, the recycled material stream comprises two main fractions: PP blended with different mineral fillers, such as talc or calcium carbonate (CaCO_3_). These fillers, originally incorporated to enhance properties such as stiffness, dimensional stability, heat resistance, and mechanical strength, remain present in the recycled material in an evaluated content of 30 wt.% [4] and significantly influence the behavior of the rPP matrix used in this study.

It should be noted that this rPP matrix originates from a mixed industrial polymer feedstock derived from two virgin polypropylene compounds used in the production line, namely POLIFOR^®^ 12 CA/40 H-D NATURALE (40 wt.% of CaCO_3_) and SYNTEGUM 1120FT NEUTRO/120 FTB NEUTRO HMFL (20 wt.% of talc).

In order to provide a complete description of the processing conditions used as a benchmark for comparison with the recycled material, Table 1 summarizes the injection moulding parameters applied to the virgin polymer materials.

#### 2.1.2. Post-Consumer Recycled Cellulose Fibers

The reinforcing material used consisted of ZZC500 cellulose fibers (ARBOCEL^®^), obtained from post-consumer waste streams and supplied by J. Rettenmaier and Söhne GmbH (Rosenberg, Germany). According to the technical datasheet, these fibers have a cellulose content of approximately 80%, along with an ash content (850 °C, 4 h) of around 15%.

#### 2.1.3. Coupling Agent (MAPP)

Maleic anhydride-grafted polypropylene (MAPP), Licocene^®^ PP MA 7452 GR, supplied by Clariant (Muttenz, Switzerland), was used as a compatibilizing agent to improve the interfacial adhesion between the polymer matrix and ZZC500 fibers. This additive was incorporated at a concentration of 3 wt.% in all formulations. The selection of this content is based on previous studies [18], which demonstrated that this proportion optimizes fiber–matrix adhesion and enhances material performance without negatively affecting processability.

### 2.2. Formulation Design

The formulations of the rPP, ZZC500, and MAPP compounds were prepared considering different fiber loadings (10, 20, and 30 wt.%). The objective was to analyze the influence of the reinforcement content on the material properties. The composite material formulation was developed through collaboration between GORENJE and TECOS (Celje, Slovenia), with the aim of optimizing the integration of rPP with natural fiber reinforcement. Table 2 shows the composition of each of the samples.

It is important to note that, although benchmark data from the original virgin polypropylene compounds (POLIFOR^®^ and SYNTEGUM) have been incorporated into this study to provide industrial context and reference values, the experimental design was primarily intended to evaluate the effect of recycled fiber incorporation within a recycled polypropylene matrix. Therefore, the main reference material used for comparison was the unreinforced recycled polypropylene (rPP), which allows a more consistent assessment of the reinforcement effect under identical processing conditions. This approach facilitates comparison with the virgin industrial formulations from which the recycled feedstock originated.

### 2.3. Preparation Methodology: Pre-Treatment, Mixing and Compounding

The milled rPP material was classified to achieve a homogeneous particle size distribution, targeting flakes in the range of approximately 3–5 mm, while removing both excessively coarse and fine powder fractions. This pre-treatment was a key to ensuring the material stability during the extrusion process. Subsequently, the classified rPP was gravimetrically blended with ZZC500 fibers and MAPP additive in order to achieve a proper dispersion of the materials during the twin-screw extrusion process.

**Figure 1:**
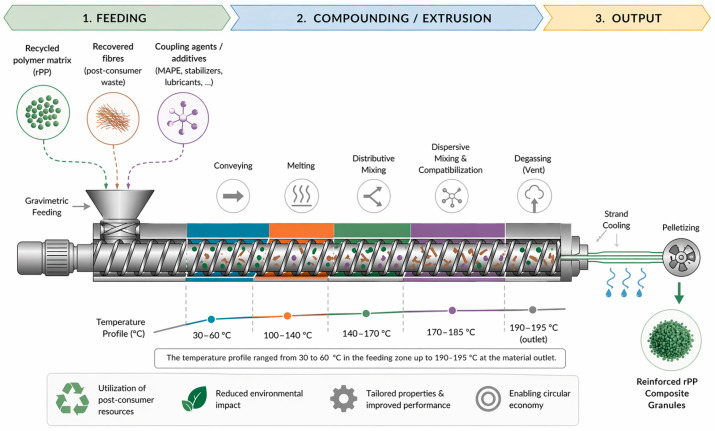
Twin extrusion compounding diagram.

The extrusion compounding process (Figure 1) was carried out using a USEON LAB-30 co-rotating twin-screw extruder (Jiangsu Yue Sheng Ji Chu Machinery Co., Ltd., Zhenjiang, China) with a length-to-diameter (L/D) ratio of 44. The operating conditions were adjusted to ensure adequate dispersion of the fibers within the matrix. The screw speed was set at 150 rpm for the lower content formulation (rPP+ZZC500-10), whereas for the higher contents (rPP+ZZC500-20 and rPP+ZZC500-30) it was reduced to 100 rpm. On the other hand, the temperature varied along the different zones of the extruder to facilitate conveying, melting, mixing, and extrusion processes. The temperature profile ranged from 30 to 60 °C in the feeding zone up to 190–195 °C at the material outlet.

After the extrusion process, all formulations were subjected to a drying process for 12 h at 60 °C. Additionally, the rPP+ZZC500-10, rPP+ZZC500-20, and rPP+ZZC500-30 formulations were further dried at 80 °C for 60, 90, and 120 min, respectively, before the injection trials.

However, at a fiber content of 30 wt.%, significant processing difficulties were observed. The compounding process could no longer be operated in an automated mode, requiring manual feeding, which was frequently interrupted due to hopper clogging.

Considering that the primary objective of this study was to assess the material’s feasibility under large-scale industrial conditions, the analysis was therefore focused on formulations with lower fiber contents (rPP+ZZC500-10 and rPP+ZZC500-20). These compositions, owing to their reduced filler content, enabled stable processing and full process automation.

### 2.4. Characterization Analysis

#### 2.4.1. Physico-Mechanical Fiber Properties

The evaluation of the physico-mechanical properties of the fibers involved the analysis of key parameters such as pH, bulk density, zeta potential, the measurement of fiber thickness and length and tensile properties.

The methodology developed for the determination of bulk density (ρ), in accordance with UNE-EN ISO 60:2024 [19], is based on measuring the density of materials capable of flowing through a standardized funnel. As established by the standard, this type of test is suitable for determining the mass per unit volume of materials in powder or granular form. The procedure consists of vertically positioning a standardized funnel through which a previously defined sample volume is poured.

Regarding pH determination, the methodology used is based on dispersing the fibers in a known volume of distilled water, followed by agitation using a magnetic stirrer (model 690-1, Nahita, Auxilab S.L., Beriáin, Spain) at 1000 rpm. The pH measurement was carried out using a pH meter (Edge, HANNA, Eibar, Spain) in accordance with UNE-ISO 6588-1 [20].

For the determination of fiber length and thickness, the methodology was based on the use of a measurement tool via scanning electron microscopy (SEM), employing a MERLIN instrument (Carl Zeiss, Zeiss GmbH, Jena, Germany), equipped with a high-resolution secondary electron detector and an integrated measurement tool.

On the other hand, the zeta potential (ZP) was determined using a Zetasizer Nano ZS (Malvern, Worcestershire, UK), which, in addition to this parameter, provides information on the electrophoretic mobility of the fibers and the conductivity of the medium.

#### 2.4.2. Mechanical Properties

Tensile testing of the material specimens was performed in accordance with ISO 527-1:2020 [21] and UNE-EN ISO 527-2:2026 [22] standards using a Beiyue WDW-10 Universal Testing Machine (ZwickRoell, Ulm-Einsingen, Germany) equipped with a 10 kN load cell and an extensometer for precise strain measurement. The following mechanical parameters were determined: Young’s modulus, tensile strength, and elongation at break. A total of 10 specimens were tested for each material condition, and the results are reported as average values together with the corresponding standard deviations.

Impact resistance was evaluated using the unnotched Charpy impact method in accordance with DIN EN ISO 179-1:2023-10 [23] to determine the energy absorption capability of the materials under dynamic loading conditions. The impact strength values are reported as average values with the corresponding standard deviations, based on measurements performed on six specimens for each material formulation (*n* = 6). All specimens were conditioned and tested in accordance with the relevant standard procedures to ensure reproducibility and comparability of the results.

#### 2.4.3. Thermal Properties

The determination of the heat deflection temperature (HDT) was carried out in accordance with UNE-EN ISO 75-2:2013 [24] (equivalent to ASTM D648 [25]), using a CEAST 20487 device (Instron, Norwood, MA, USA). The test consists of subjecting the specimen to a constant stress of 1.8 MPa while the temperature is increased at a controlled rate of 120 °C/h. The HDT is defined as the temperature at which the deformation reaches 0.2%. Three replicates were tested in order to evaluate possible deviations between measurements.

The Vicat softening temperature was determined following UNE-EN ISO 306:2023 [26] using a CEAST 20491 device (Instron, Norwood, MA, USA). The test consists of measuring the temperature at which a standardized indenter, under a load of 50 N, penetrates 1 mm into the surface of the specimen while the temperature is increased uniformly. Three replicates were also performed to assess possible deviations in the results.

#### 2.4.4. Rheological Properties

The rheological behavior was evaluated using a Göttfert Rheograph 25 capillary rheometer (Göttfert, Werkstoff-Prüfmaschinen GmbH, Buchen, Germany) under controlled testing conditions. The equipment used is equipped with a barrel diameter of 15 mm and capillaries with length-to-diameter (L/D) ratios of 10:1 and 30:1.

The tests were carried out at a constant temperature of 180 °C, applying a shear rate range from 10 s^−1^ to 10,000 s^−1^. These conditions allow the characterization of material flow over a wide range representative of processing techniques such as injection moulding. Three replicates were tested in order to evaluate possible deviations between measurements.

### 2.5. Industrial-Scale Validation

With the aim of evaluating the feasibility of the developed material under real production conditions, injection moulding trials were carried out in a total of seven companies: four companies located in Andalusia (Spain) and three companies based in Slovenia. This multi-site validation, coordinated by ECOCASTULUM (Linares, Jaén, Spain) and TECOS, made it possible to analyze the material’s injectability in different production environments, with varying machine configurations and mould geometries.

The trials were conducted using the injection moulding equipment available at each facility, maintaining the standard operating conditions of each company in all cases. It is important to highlight that no modifications to the equipment or production lines were required to process the material, demonstrating its compatibility with standard industrial infrastructures.

The main processing parameters employed during the industrial injection moulding trials are summarized in Table 3. The reported conditions include injection temperature, mould temperature, injection speed, injection and holding pressure and cooling time. Although slight adjustments associated with the geometry of the injected parts and the operational characteristics of each industrial machine were required, all trials were successfully carried out under conventional industrial operating conditions using standard processing windows for polypropylene-based materials.

The participating companies, their locations, and the equipment used in each case are detailed below:

- PLASTITEC (Bedmar, Jaén, Andalusia, Spain)—Injection moulding machine: BOLE 100 EKS/C340.
- PLASTBAEZ (Guarromán, Jaén, Andalusia, Spain)—Injection moulding machine: ENGEL 225 TN.
- INYECCEP (Villa del Río, Córdoba, Andalusia, Spain)—Injection moulding machine: PROTECNOS PTX 170.
- SESÉ INTEGRA (Martos, Jaén, Andalusia, Spain)—Injection moulding machine: Negri Bossi 1300 TN.
- ADRIA MOBIL (Novo Mesto, Slovenia)—Injection moulding machine: KRAUSS MAFFEI 80/380 CX.
- IMP PUMPS (Komenda, Slovenia)—Injection moulding machine: FANUC ROBOSHOT ALPHA S50IA/330/IT.
- MDCN TECH (Ljubljana, Slovenia)—Injection moulding machine: TOYO Innovex Si 80-7.

## 3. Results and Discussions

### 3.1. Physico-Mechanical Fiber Properties

The pH of the ZZC500 fibers was 7.88, indicating a slightly basic surface behavior. This parameter is directly related to the surface chemistry of the fibers and may influence their charge as well as their chemical interactions with the polymer matrix [27]. The measured bulk density was 99.65 g/L, which is lower compared to other natural fibers reported in the literature [28,29].

The zeta potential of the fibers was −10.900 mV, while the electrophoretic mobility was measured at −0.855 µm·cm/V·s, indicating a negative surface charge that may enhance dispersion stability and interfacial compatibility with the polymer matrix. The recorded electrical conductivity was 0.094 mS/cm.

Regarding dimensions, the fiber length ranged from 129.2 µm to 11.79 mm, while the thickness varied between 8.21 µm and 33.65 µm. These values have a direct impact on the mechanical reinforcement capability of the composites and on their processability during extrusion and injection moulding.

### 3.2. Thermal Properties

Unreinforced rPP exhibited a Vicat temperature of 97 °C. When compared with the available data for the virgin benchmark materials, this value falls within the range reported for POLIFOR^®^ 12 CA/40 H-D NATURALE (92–100 °C), which is a calcium carbonate-filled PP compound, indicating that the recycled matrix retains a thermal softening behavior comparable to conventional mineral-filled polypropylene systems. For SYNTEGUM 1120FT NEUTRO/120 FTB NEUTRO HMFL, not publicly available, the Vicat temperature is specified in the technical documentation.

The incorporation of fibers led to an overall increase in this temperature across all formulations. In the case of composites with ZZC500 fiber, increasing the fiber content from 10% to 20% resulted in a rise in the Vicat temperature from 109 °C to 113 °C.

Overall, the results indicate that the addition of fibers contributes to improving the thermal resistance of rPP by increasing its softening temperature. This effect becomes more pronounced as the reinforcement content increases, suggesting greater suitability of these materials for applications requiring thermal stability at elevated temperatures.

When benchmarked against the virgin reference materials, the recycled systems exhibit equal or superior Vicat softening temperatures compared to the CaCO_3_-filled virgin PP (POLIFOR^®^). This indicates that the combination of the recycled mineral-filled matrix and the additional reinforcement led to a synergistic improvement in thermal performance.

Table 4 shows the Vicat temperature for virgin PP, rPP, rPP+ZZC500-10, and rPP+ZZC500-20 samples.

Regarding HDT results, rPP exhibited a temperature of 64 °C. When compared with the virgin reference material POLIFOR^®^ 12 CA/40 H-D NATURALE, which shows an HDT value of approximately 120 °C, the recycled matrix presents a significantly lower resistance to thermal deformation under load. The incorporation of fibers led to an overall improvement in resistance to thermal deformation. In ZZC500 fiber-reinforced composites, a more significant increase was observed, from 69 °C to 75 °C.

Taken together, the results (Table 5) show that fiber addition enhances the dimensional stability of the material under combined temperature and load conditions, with this effect being more evident in formulations with reinforcement contents around 20 wt.%.

### 3.3. Mechanical Properties

A clear influence of the ZZC500 mineral reinforcement on the mechanical behavior of rPP can be observed (Table 6). POLIFOR^®^ exhibits a markedly different balance of properties, combining very high ductility (elongation at break 60%) and impact resistance (50 kJ/m^2^) with a stiffness of around 3400 MPa, which is significantly higher than that of the rPP-based formulations. By contrast, the recycled materials show a clear shift toward a more rigid and less deformable behavior, with substantially lower elongation and impact performance, as a consequence of their heterogeneous recycled structure and inherent mineral content. In the case of SYNTEGUM 1120FT NEUTRO/120 FTB NEUTRO HMFL, no mechanical data are available in the technical documentation.

Regarding stiffness, the tensile elastic modulus (Et) increases progressively with filler content, rising from 2414 ± 58 MPa to 2886 ± 49 MPa for the material with 20% loading. This trend confirms the effect of the reinforcement.

In terms of tensile strength (σM), a significant improvement is also observed with filler incorporation. This behavior suggests good matrix–filler interaction, which promotes efficient stress transfer and leads to an overall enhancement of the mechanical strength of the material.

On the other hand, the elongation at break (εt) decreases with increasing filler content, dropping from 3.7 ± 1% for rPP to 3.3 ± 0.5% and 2.2 ± 0.4% for the composites with 10% and 20% ZZC500, respectively. This trend is typical of systems reinforced with rigid fillers, where improvements in stiffness and strength are accompanied by a reduction in ductility.

Finally, the impact behavior shows a non-linear response. While the incorporation of 10% ZZC500 slightly reduces the impact resistance (9.8 ± 0.6 kJ/m^2^ compared to 10.6 ± 0.5 kJ/m^2^ for rPP), increasing the filler content to 20% leads to a significant improvement, reaching 12.9 ± 0.7 kJ/m^2^.

### 3.4. Rheological Properties

From a processing standpoint, the 20% fiber content allowed stable operating conditions to be maintained during the dosing, drying, and pelletizing stages, ensuring its feasibility at an industrial scale. Consequently, this formulation was selected for industrial-scale validation, as well as for capillary rheological analysis, with the aim of evaluating the material’s rheometric behavior.

To obtain viscosity values, the Bagley correction was applied, allowing the determination of the true shear stress and real shear rate from the experimental results obtained in capillary rheology. Experimental data under flow instability were excluded from the Bagley correction.

Table 7 shows the shear viscosity of the rPP+ZZO500-20 sample.

**Figure 2:**
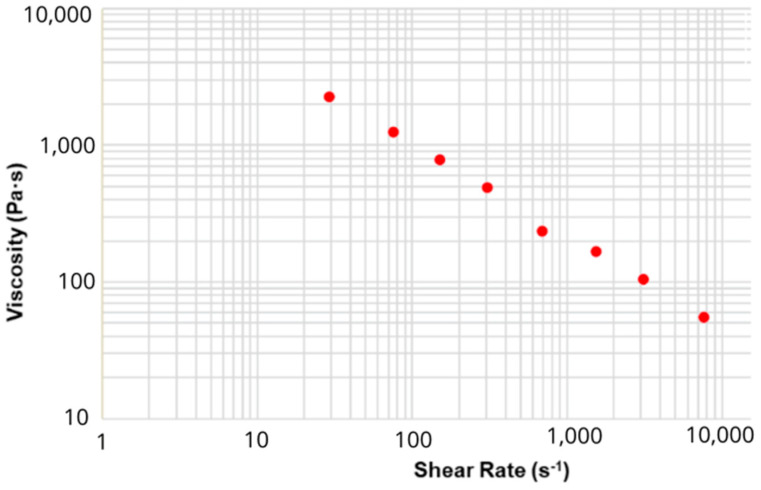
Viscosity curve of rPP+ZZ500-20 at 180 °C.

Figure 2 shows the corrected viscosity results at 180 °C.

### 3.5. Industrial-Scale Validation

Based on the previously obtained results, as well as considering the material’s processability, the rPP+ZZC500-20 formulation was selected for industrial scale-up. This choice was primarily based on its environmental advantages, as it incorporates post-consumer recycled cellulose, thereby contributing to the principles of circular economy and waste valorization.

In addition, this formulation exhibited suitable mechanical performance (Table 6), demonstrating good interfacial adhesion between the fiber and the matrix, along with significant improvements in crystallinity and thermal stability.

In the different replication cases, parts with diverse geometries and functional requirements were manufactured, representative of real industrial applications. This variety of morphologies made it possible to observe the material’s behavior under different mould injection conditions, cooling rates, and other critical aspects of the injection moulding process.

The industrial validation activities carried out in the present study were conceived as feasibility trials under real manufacturing conditions rather than as controlled statistical production campaigns. Consequently, the participating companies operated using their standard industrial configurations, processing parameters and production routines, with the primary objective of evaluating the capability of the developed material to be processed using conventional injection moulding equipment without requiring modifications to the machinery or molds. For this reason, quantitative production indicators such as reject rate, dimensional deviations, part mass variability or defect frequency were not systematically monitored during the replication activities. Nevertheless, the successful processing of the material in different industrial environments demonstrated its operational compatibility and industrial applicability under standard manufacturing conditions.

**Figure 3:**
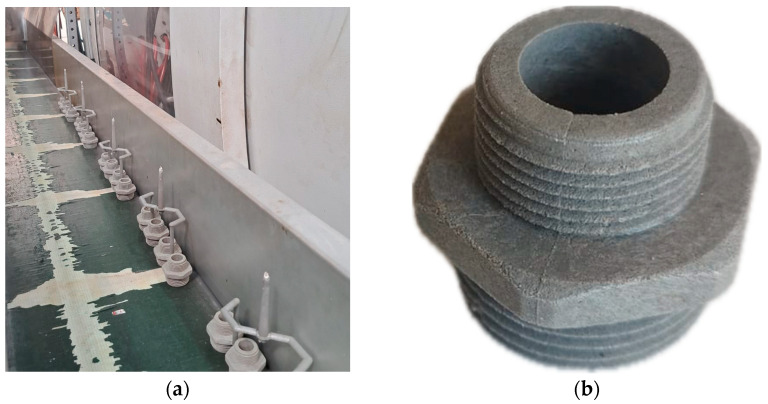
Plastitec’s replication case: (**a**) Injection process and (**b**) irrigation valve.

In the case of the company PLASTITEC, the material was processed by injection moulding for the production of an irrigation valve intended for the agricultural sector. This application represents a functional component with specific requirements in terms of geometry. As shown in Figure 3, the material exhibited appropriate behavior during the injection process, adapting to standard operating conditions and enabling the production of parts in accordance with the required geometry. Furthermore, following quality control carried out at the company, no significant differences were observed compared to parts manufactured using virgin PP.

**Figure 4:**
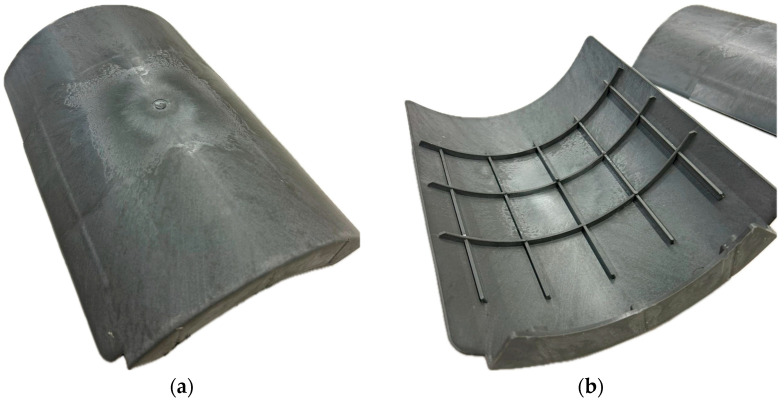
Plastbaez’s replication case: (**a**) Corner bed and (**b**) corner bed back.

The second industrial validation was carried out at PLASTBAEZ. In this case, the developed material was tested in the injection moulding of corner fittings used in bed bases (Figure 4). The resulting part exhibited a surface finish with slight variations in material color. However, no issues were recorded during the injection process compared to the material typically processed in this mold.

**Figure 5:**
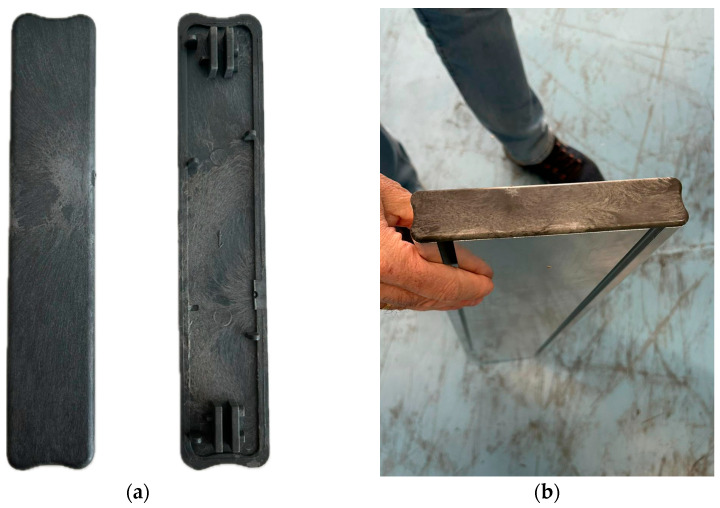
Inyeccep’s replication case: (**a**) Injected parts for the train seats and (**b**) piece placed on train seats.

The third industrial implementation, carried out at INYECCEP, marked a significant step forward in terms of sector diversification. The selected component was related to the mobility sector. The injected part corresponded to a decorative cladding element used in train seat assemblies (Figure 5a).

The injection trials confirmed stable processing behavior and a satisfactory surface finish, which is critical for visible components. Figure 5b shows the manufactured part assembled onto the original train seat structure.

**Figure 6:**
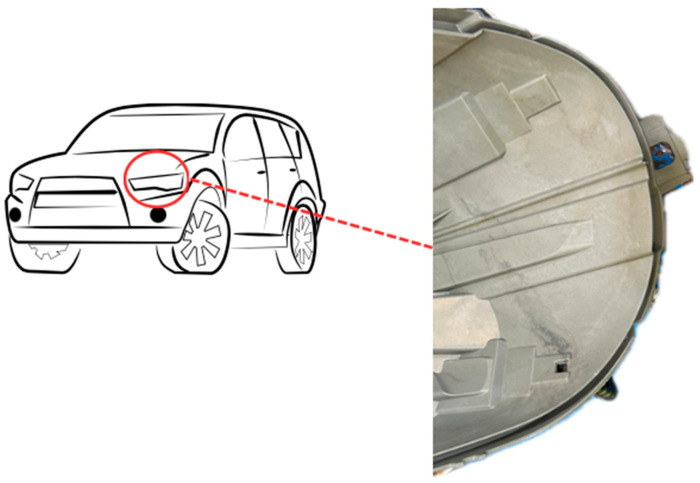
Sesé Integra’s replication case.

The fourth replication case was carried out at SESÉ Integra facilities, within the automotive sector. In this case, the developed material was processed through injection moulding using an industrial mould provided by a well-known automotive manufacturer. The demonstrator corresponds to a component of a car headlight (Figure 6). The successful processing of the material under these conditions highlights its suitability for high-value industrial applications. Due to confidentiality constraints associated with the industrial partner, only a partial view of the final component can be disclosed.

The transferability of the developed material across different industrial sectors was also validated through three independent replicative case studies conducted in Slovenia.

**Figure 7:**
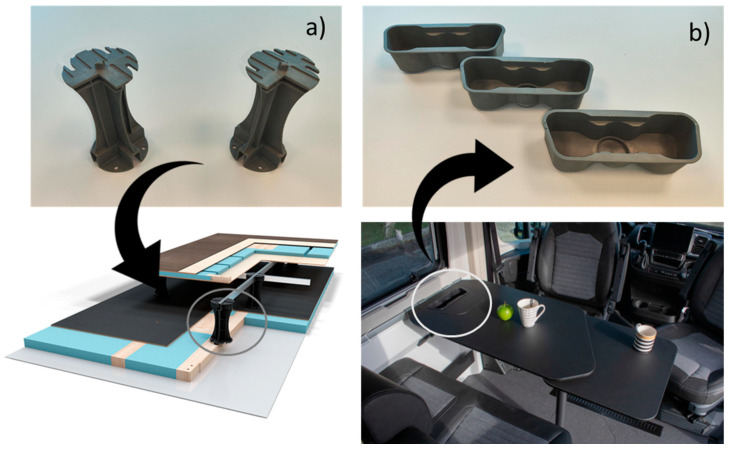
ADRIA Mobil’s replication case: (**a**) Distance holder for Motorhome’s double-floor decking and (**b**) cup holder compartment in the interior design of RVs.

In the case of ADRIA MOBIL, a leading manufacturer in the recreational vehicle (RV) sector, the material was processed by injection moulding for two interior components: a structural distance holder used in the double-floor system of the motorhomes and a cup holder integrated into a foldable table (Figure 7). Both applications represent functional parts with specific requirements in terms of dimensional stability and surface quality. The material exhibited stable processing behavior under standard operating conditions, without the need for process adjustments. The resulting components met the required quality criteria, with no significant deviations compared to conventionally used materials.

**Figure 8:**
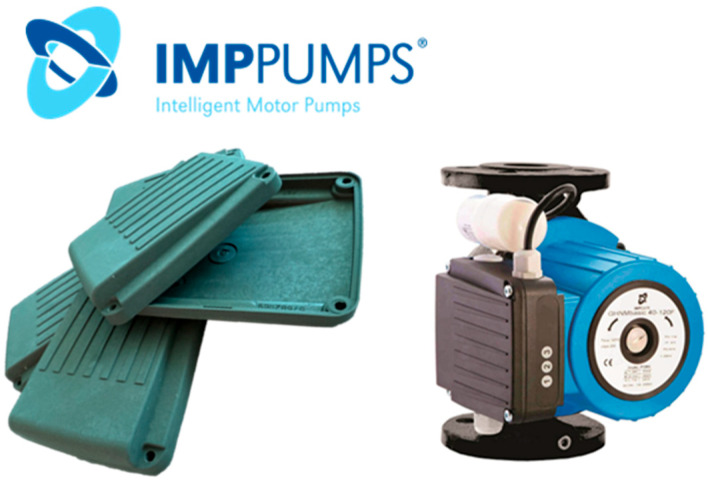
IMP Pump’s housing is made of composite materials for the electronic system of the water pump.

The second industrial validation was carried out at IMP Pumps Ltd., where the developed composite was applied in the injection moulding of housings for electrical components in water pump systems (Figure 8). This application represents a technically relevant component, requiring adequate dimensional stability and reliable processing behavior. The material was processed under identical conditions as the reference material, without the need for modifications to the injection moulding parameters. During processing, the composite exhibited stable flow characteristics and good mouldability, enabling consistent cavity filling and part formation. The resulting components showed high surface quality, good dimensional accuracy, and sufficient structural integrity, confirming the material’s suitability for use in electromechanical applications.

**Figure 9:**
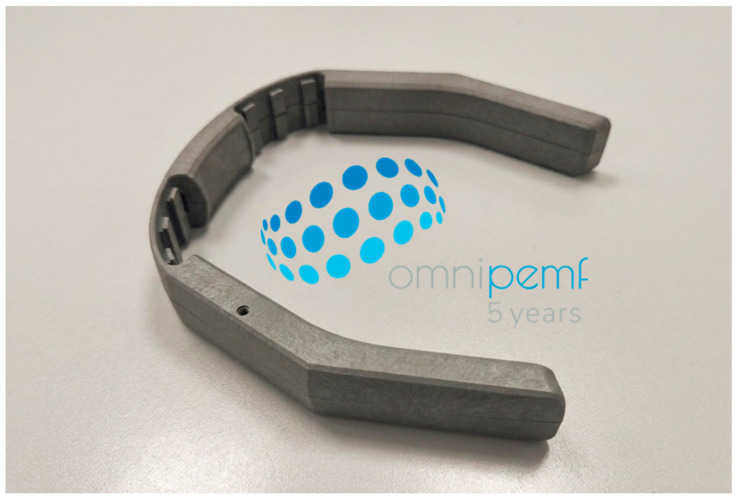
Housing component for NeoRhythm PEMF wearable device (MDCN Tech company).

The third case study was performed at MDCN Tech company, involving the production of injection-molded housings for the NeoRhythm wearable device (Figure 9). NeoRhythm is a non-invasive, science-based consumer health device that utilizes pulsed electromagnetic field (PEMF) technology to stimulate the vagus nerve, supporting improved sleep quality, stress reduction, and overall physiological balance. The product integrates advanced electronics within a compact, ergonomically designed housing, requiring both high functional reliability and a high-quality surface finish.

The developed composite material was successfully processed under standard injection moulding conditions, demonstrating stable flow behavior and good moldability. The resulting housings met the required dimensional tolerances and exhibited satisfactory surface quality, confirming their suitability for consumer-facing applications. This case study highlights the applicability of the developed materials in high-value products where both functional performance and esthetic appearance are critical.

It is worth highlighting that, regardless of the company, the type of part, or the equipment used, the material could be processed under standard production conditions, without the need for machine adjustments or modifications to the molds compared to those used for conventional materials. The adaptability of the developed material across the different industrial environments analyzed demonstrates its robustness and compatibility with existing industrial processes.

These results indicate that the rPP composite reinforced with ZZC500 fiber exhibits sufficient stability to be considered technically viable for application in different sectors through injection moulding. The multi-site validation approach confirms that the material’s performance is not limited to a single controlled configuration, thereby supporting its scalability and the increase in its TRL.

## 4. Conclusions

This research demonstrates the technical feasibility of the material developed from rPP, ZZC-500, and MAPP, and confirms the reproducibility of the material across different injection moulding systems, from laboratory scale to industrial scale. The main conclusions are highlighted below:

- During the development of the rPP+ZZC500-20 composite, it was confirmed that the addition of 20% fibers improved processability in extrusion, pelletizing, and injection moulding without the need for modifications to industrial equipment.
- The incorporation of ZZC500 fibers increased the Vicat softening temperature and the heat deflection temperature (HDT) of the rPP matrix, with higher fiber contents providing greater thermal stability.
- Multi-site industrial validation in Spain and Slovenia confirmed the technical feasibility of the material for different applications, part geometries, and injection moulding equipment.
- The incorporation of post-consumer ZZC-500 cellulose fibers contributes to circular economy principles through waste valorization while maintaining adequate processing and thermal behavior for industrial injection moulding applications.

Based on the obtained results, the rPP composite reinforced with ZZC500 fibers demonstrated stable industrial processability together with improved thermal behavior. Therefore, the development of this material represents a technically and environmentally viable alternative for the production of industrial parts through injection moulding.

## Figures and Tables

**Table 1:** Injection moulding conditions of the virgin material during the industrial injection moulding trials.

Sample	Mould Temperature (°C)	Injection Temperature (°C)	Injection Speed (mm/s)	Injection Pressure (bar)	Holding Pressure (bar)	Holding Time (s)
POLIFOR^®^ 12 CA/40 H-D NATURALE	50	220	80	500	450	20
SYNTEGUM 1120FT NEUTRO/120 FTB NEUTRO HMFL	30	245	60	500–600	400	20

**Table 2:** Sample composition of rPP, rPP+ZZC500-10, rPP+ZZC500-20 and rPP+ZZC500-30 composites.

Sample Formulation	rPP (wt.%)	ZZC500 (wt.%)	MAPP (wt.%)
rPP	100	0	0
rPP+ZZC500-10	87	10	3
rPP+ZZC500-20	77	20	3
rPP+ZZC500-30	67	30	3

**Table 3:** Injection moulding conditions during the industrial injection moulding trials.

Case	Mould Temperature (°C)	Injection Temperature (°C)	Injection Speed (mm/s)	Injection Pressure (bar)	Holding Pressure (bar)	Holding Time (s)
Plastitec	20	180	90	500	510	20
Plastbáez	20	180	60	650	550	25
Inyeccep	20	185	80	900	700	30
Sesé Integra	25	180	90	900	750	35
Adria Mobil	20	190	80	500	300	20
IMP Pumps	25	190	100	650	400	25
MDCN Tech	20	180	60	600	480	25

**Table 4:** Vicat softening temperature of virgin PP, rPP, rPP+ZZC500-10 and rPP+ZZC500-20 composites.

Sample Formulation	VICAT Softening Temperature (°C)
POLIFOR^®^ 12 CA/40 H-D NATURALE	92–100
SYNTEGUM 1120FT NEUTRO/120 FTB NEUTRO HMFL	na ^1^
rPP	97.2 ± 0.001
rPP+ZZC500-10	109.0 ± 0.010
rPP+ZZC500-20	113.0 ± 0.021

^1^. Not publicy available.

**Table 5:** HDT of rPP, rPP+ZZC500-10 and rPP+ZZC500-20 composites.

Sample Formulation	HDT (°C)
POLIFOR^®^ 12 CA/40 H-D NATURALE	120
SYNTEGUM 1120FT NEUTRO/120 FTB NEUTRO HMFL	na ^1^
rPP	64.0 ± 0.0005
rPP+ZZC500-10	69.0 ± 0.0012
rPP+ZZC500-20	75.0 ± 0.019

^1^. Not publicy available.

**Table 6:** Tensile parameters of rPP and rPP+ZZC500-20 composites.

Sample Formulation	E_t_ (MPa)	σ_M_ (MPa)	ε_t_ (%)	Impact Resistance (KJ/m^2^)
POLIFOR^®^ 12 CA/40 H-D NATURALE	3400	22	60	50
SYNTEGUM 1120FT NEUTRO/120 FTB NEUTRO HMFL	na ^1^	na ^1^	na ^1^	na ^1^
rPP	2414 ± 58	21.8 ± 0.8	3.7 ± 1	10.6 ± 0.5
rPP+ZZC500-10	2618 ± 39	26.5 ± 1.2	3.3 ± 0.5	9.8 ± 0.6
rPP+ZZC500-20	2886 ± 49	28.5 ± 0.6	2.2 ± 0.4	12.9 ± 0.7

^1^. Not publicy available.

**Table 7:** Shear viscosity: Experimental results at 180 °C.

Share Rate (γ) (s^−1^)	Viscosity (η)
29.1	2250 ± 45
75.4	1250 ± 32
152	783 ± 21
304	490 ± 15
686	235 ± 9
1550	167 ± 6
3130	106 ± 4
7650	55.7 ± 2.1

## Data Availability

The original contributions presented in this study are included in the article. Further inquiries can be directed to the corresponding author.

## Abbreviations

The following abbreviations are used in this manuscript:

PP	Polypropylene
rPP	Recycled polypropylene
MAPP	Maleic anhydride-grafted polypropylene
PP-GF	Glass fiber-reinforced PP
HDT	Heat deflection temperature
ZP	Zeta potential
L/D	Length-to-diameter
SEM	Scanning electron microscopy
TRL	Technological readiness level

## References

[1] Srivastav R.S., More A.P. (2026). Advanced techniques and mechanistic insights for high efficiency recovery of polymers recycling. J. Environ. Manag..

[2] Wang Z., Fang E., Chen L., Fan Z., Song S. (2026). Recent developments in recycling technologies for polymeric plastics. Polym. Chem..

[3] Badini C., Ostrovskaya O., Bernagozzi G., Lanfranco R., Miranda S. (2023). Recycling of Polypropylene Recovered from a Composting Plant: Mechanical Behavior of Compounds with Virgin Plastic. Recycling.

[4] Žepič Bogataj V., Martínez García C., Cotes Palomino M.T., López A.B., Mezek B., Kotnik S. (2026). Recycled Sisal Fiber-Reinforced Polypropylene Derived from Industrial Waste Stream. Detritus.

[5] Gavilanes D., Valle V., Quiroz F., Cadena F., Iribarren J.I. (2025). Valorizing urban pruning wastes and recycled polyethylene towards sustainable natural fiber-reinforced polymer composites. Clean. Mater..

[6] Das S.C., la Rosa A.D., Goutianos S., Grammatikos S. (2023). Effect of accelerated weathering on the performance of natural fibre reinforced recyclable polymer composites and comparison with conventional composites. Compos. Part C Open Access.

[7] Le Lee K.J., Tan K.H. (2025). Effects of hybrid recycled polypropylene and natural kenaf fibres on spalling prevention of ultra-high performance concrete at elevated temperatures. Cem. Concr. Compos..

[8] Alhakim G., Jaber L. (2025). Mechanical behaviour of polypropylene–sand composites reinforced with natural fibres. Proc. Inst. Civ. Eng. Ground Improv..

[9] Espinach F.X., Vilaseca F., Tarrés Q., Delgado-Aguilar M., Aguado R.J., Mutjé P. (2024). An alternative method to evaluate the micromechanics tensile strength properties of natural fiber strand reinforced polyolefin composites. The case of hemp strand-reinforced polypropylene. Compos. Part B Eng..

[10] Arinze R.U., Oramah E., Chukwuma E.C., Okoye N.H., Eboatu A.N., Udeozo P.I., Chris-Okafor P.U., Ekwunife M.C. (2023). Reinforcement of polypropylene with natural fibers: Mitigation of environmental pollution. Environ. Chall..

[11] Houben S., Membrado M.M., van Belleghem L., Olazabal I., van Velthoven N., Vanbroekhoven K., Sardon H., de Vos D., Feghali E., Elst K. (2025). Chemical recycling of nitrogen containing polymers: Processes and industrial prospects. Prog. Polym. Sci..

[12] De B., Bera M., Bhattacharjee D., Ray B.C., Mukherjee S. (2024). A comprehensive review on fiber-reinforced polymer composites: Raw materials to applications, recycling, and waste management. Prog. Mater. Sci..

[13] Nascimento H.M., Granzotto D.C.T., Radovanovic E., Fávaro S.L. (2021). Obtention and characterization of polypropylene composites reinforced with new natural fibers from *Yucca aloifolia* L. Compos. Part B Eng..

[14] Mahesh A., Rudresh B.M., Reddappa H.N. (2022). Potential of natural fibers in the modification of mechanical behavior of polypropylene hybrid composites. Mater. Today Proc..

[15] Colledani M., Bonaiti G., Cortinovis F., Diani M. (2026). Production quality control of mechanical recycling systems for specification-compliant circular manufacturing of fiber-reinforced polymer products. CIRP Ann..

[16] Hubbard A.M., Gelfond J., Slavny K., Kooduvalli K., Copenhaver K., Lamm M.E., Wasti S., Korey M., Pu Y., Clarkson C.M. (2025). Elucidating the impact of fiber source on polypropylene/hemp composite performance for the automotive industry. Mater. Adv..

[17] Ichim M., Muresan E.I., Lisa G., Ciolacu F., Puițel A.C. (2025). Valorisation of Recycled Cotton as Reinforcement in Recycled Polypropylene Composites. Textiles.

[18] Zepič Bogataj V., Fajs P., Peñalva C., Omahen M., Čop M., Henttonen A. (2019). Utilization of recycled polypropylene, cellulose and newsprint fibres for production of green composites. Detritus.

[19] (2024). Plastics-Determination of the Bulk Density of Materials Susceptible to Pass Through a Given Funnel.

[20] (2026). Paper, Board and Pulps. Determination of pH of Aqueous Extracts. Part 1: Cold Extraction.

[21] (2020). Plastics-Determination of Tensile Properties-Part 1: General Principles.

[22] Plastics. Determination of Tensile Properties. Part 2: Test Conditions for Plastics for Moulding and Extrusion. 2026. Tienda AENOR. https://tienda.aenor.com/p/norma-une-en-iso-527-2-2026-n0075564.

[23] Plastics—Determination of Charpy Impact Properties—Part 1: Non-Instrumented Impact Test. 2023. Tienda AENOR. https://tienda.aenor.com/p/norma-din-en-iso-179-1-2023-10-369770968.

[24] (2013). Plastics-Determination of Temperature of Deflection Under Load-Part 2: Plastics and Ebonite.

[25] (2018). Standard Test Method for Deflection Temperature of Plastics Under Flexural Load in the Edgewise Position. https://store.astm.org/d0648-18.html.

[26] (2022). Plastics-Thermoplastic Materials-Determination of Vicat Softening Temperature (VST).

[27] Lee C.H., Khalina A., Lee S.H. (2021). Importance of Interfacial Adhesion Condition on Characterization of Plant-Fiber-Reinforced Polymer Composites: A Review. Polymers.

[28] Álvarez M., Marrocco T., Sillars F. (2026). Engineered gypsum composites with superabsorbent-polymers and natural/recycled fibre reinforcement: Multi-scale performance and validation. Constr. Build. Mater..

[29] Sayam S. (2026). Natural fibers in sustainable materials: Extraction technologies, fiber modification, and performance–sustainability relationships. RSC Adv..

